# Feasibility and outcomes of single-incision robotic nipple-sparing mastectomy: a systematic review and meta-analysis

**DOI:** 10.1007/s11701-026-03297-6

**Published:** 2026-03-11

**Authors:** Hasan Asfour, Hussein Lubbad, Ewa Anna Sobczak, Walid Sasi

**Affiliations:** 1 Leicester Cancer Research Centre, Department of Genetics, Genomics & Cancer Sciences, University of Leicester, Clinical Sciences Building, Leicester Royal Infirmary, LE1 5WW, Leicester, UK; 2 Department of Breast Surgery, University Hospitals of Leicester NHS Trust, Leicester, UK; 3 Department of General Surgery, University Hospitals of Leicester NHS Trust, Leicester, UK; 4 Department of Breast Surgery, Wrightington, Wigan and Leigh NHS Teaching Hospitals Foundation Trust, Wigan, Lancashire UK

**Keywords:** Breast surgery, Complication, Mastectomy, Minimally invasive surgery, NSM, Robotic surgery

## Abstract

Single-incision robotic nipple-sparing mastectomy (RNSM) is an emerging minimally invasive approach for breast cancer and prophylactic surgery, yet its safety and feasibility remain to be confirmed. We conducted a comprehensive systematic review and meta-analysis to examine the single-incision RNSM perioperative parameters and complications. A bibliographic search was conducted according to the PRISMA guidelines across four databases. Following deduplication and exclusion, thirteen studies were eligible and constituted this study. Data were combined using random-effects models. Forest plots were generated to estimate pooled proportions using the Freeman-Tukey double arcsine transformation, and heterogeneity was assessed via I^2^ statistics. Quality assessment was performed using the Joanna Briggs Institute Checklist for Prevalence Studies. Funnel plots were used to assess publication bias. This study was registered on the PROSPERO registry. The pooled mean operative time was 273 min, and the mean blood loss was 82 ml. Pooled complication rates were relatively low, including skin-flap necrosis (2.4%), nipple-areolar complex necrosis (0.4%), delayed wound healing (4.7%), surgical-site infection (3.8%), deep infection (1.2%), hematoma (2.4%), and seroma (5.8%). Pooled estimate of positive margin rate was < 1%. Locoregional and distant recurrence rates were 0.8% and 0.4%, respectively. No conversions to open surgery or procedure-related deaths were reported. The single-incision RNSM is a modern surgical approach with acceptable perioperative parameters and satisfactory complication rates. However, heterogeneity across studies and the relative rarity of long-term oncological data warranted cautious interpretation. Prospective comparative trials are necessary to prove the oncological safety and economic effectiveness compared with other approaches.

**Supplementary Information:**

The online version contains supplementary material available at 10.1007/s11701-026-03297-6.

## Introduction

Nipple-sparing mastectomy (NSM) is a surgical approach that aims to remove the breast tissue while preserving the overlying skin and nipple-areola complex (NAC). The NSM is considered oncologically safe for selected breast cancer patients, as well as for prophylactic mastectomy candidates [1, 2]. Preservation of the NAC in NSM allows for improved cosmetic satisfaction and psychological outcomes compared to conventional approaches, such as skin-sparing mastectomy or total mastectomy [3, 4]. However, NSM is often associated with drawbacks, including risks of ischemia and necrosis of the skin flap or NAC, along with technical limitations, such as ergonomic challenges for surgeons, especially in patients with large and ptotic breasts [5, 6].

Robotic-assisted nipple sparing mastectomy (RNSM) appeared as a minimally invasive method, enabling surgeons to conduct complex dissections through small, concealed incisions and using high definition, three-dimensional visualization and articulated instruments [7, 8]. The RNSM approach optimizes the patients’ post-operative complications and the surgeons’ workload burden [8–10]. Despite its favorable outcomes and ergonomic advantage, RNSM is still time-intensive, technically demanding, and costly when compared with conventional approaches or other minimally invasive techniques, such as endoscopic mastectomy [11–13].

The RNSM often employs a multi-port approach, permitting the passage of a camera and dissection instruments [14, 15]. This technique can be performed either with gas insufflation or gasless (using retractors) [14, 16]. Although these techniques are efficient, the requirement for multiple incisions may compromise cosmetic benefits [15]. Single-port and single-incision approaches are recent advancements in RNSM, which offer a theoretical advantage of reduced surgical trauma and improved esthetic outcome (single incision), with the maintenance of oncological and reconstructive goals of NSM [17].

Despite attractiveness and growing interest, perioperative and postoperative outcomes for single-incision RNSM remain unclear. Current evidence comes mainly from small sample-sized single-center reports with variable documentation of complications [15, 18–21]. A detailed pooling of presently available data is required to refine a better definition of perioperative variables, rates of complications, and oncological outcomes. To this end, a systematic review with meta-analysis was conducted to examine the feasibility and outcomes of single-incision RNSM by pooling results from the existing literature, offering a comprehensive and up-to-date synthesis of evidence to enhance clinical practice and direct future research endeavors.

## Methods

### Search strategy and registration

**Fig. 1:**
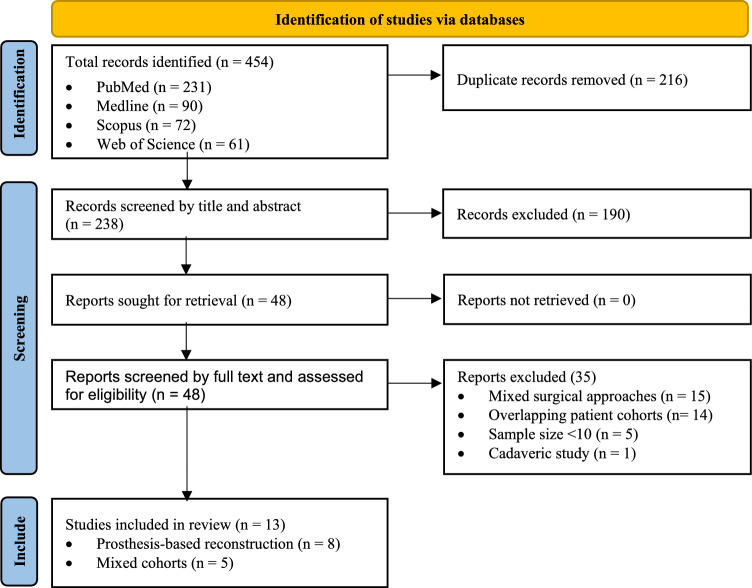
Flowchart demonstrating the systematic approach of record identification, screening, and inclusion according to PRISMA guidelines. Records were retrieved from four databases: PubMed, Medline, Scopus, and Web of Science. After duplicate removal and screening, thirteen studies were included in the review, reporting either prosthesis-based reconstruction cohorts (n = 8) or mixed cohorts (n = 5)

This comprehensive systematic review and single-arm meta-analysis study was conducted in accordance with the Preferred Reporting Items for Systematic Reviews and Meta-Analyses (PRISMA) 2020 guidelines [22] (Fig. 1). A comprehensive search was performed to identify eligible studies that reported on perioperative parameters and postoperative outcomes of single-incision RNSM. Four electronic databases, including PubMed, Medline, Scopus, and Web of Science, were searched (Fig. 1). Search strategy comprised relevant keywords linked by either of the Boolean operators (Table S1). No time restriction was applied, and a literature search was conducted up to August 12, 2025, “search date”. This study is registered on PROSPERO, an international systematic review registry platform (CRD420251157574).

### Research question and eligibility criteria

This study aimed to assess the feasibility, perioperative parameters, complication rates, and oncologic outcomes of the single-incision approach in robotic nipple-sparing mastectomy. The following inclusion criteria were used to identify eligible studies: *i*) peer-reviewed clinical studies including clinical trials, cohort studies, or case series with ≥ 10 patients; *ii*) studies reporting outcomes of NSM performed using single-port or single-incision robotic systems; *iii*) studies published in English; and *iv*) studies providing either comparative or descriptive outcome data. The excluded studies were case reports or series reporting on < 10 patients, review articles, editorial comments, letters to the editor, abstracts presented as part of a conference, studies involving cadaveric or non-human subjects, studies that did not clearly describe the single-port robotic technique, and duplicate patient cohorts. The research question and eligibility criteria were defined using the PICO (Population, Intervention, Comparator, and Outcomes) framework and are summarized in Table S1.

### Study selection and data extraction

A total of 454 records were identified through the systematic database searches. After deduplication, 238 records remained. Two reviewers independently screened titles and abstracts, resulting in 48 reports sought for full-text assessment. Following full-text review, 35 studies were excluded for the following reasons: mixed surgical approaches (n = 15), overlapping patient cohorts (n = 14), sample size < 10 (n = 5), cadaveric study (n = 1). Thirteen studies met the eligibility criteria, which were divided into two groups: prosthesis-based reconstruction cohorts (n = 8) and mixed cohorts (n = 5) (Fig. 1). Discrepancies during the selection process were resolved through discussion or, when necessary, consultation with a senior third reviewer.

Two independent reviewers extracted data pertaining to the characteristics of the included studies, like study design, sample size, follow-up duration and patients’ characteristics (Table 1 and Table 2), peri-operative variables (Table 3), and postoperative complications (including skin flap necrosis, NAC necrosis, surgical site infection (SSI), deep infection, delayed wound healing, hematoma, seroma, and cancer recurrence). Discrepancies were resolved by consensus or senior author arbitration. Zotero software (version 7.0.27) was used for reference management and duplicate removal. No artificial intelligence tools were used in the selection or extraction process.

**Table 1:** The table summarises key features of each study reporting single-incision RNSM followed by prosthesis-based reconstruction. BMI: body mass index; NSM: nipple-sparing mastectomy; N/A: not available; y: year; cm: centimeter; g: gram; m: month

Study	Location	Study design	Patients’ number	Age (y)	BMI	NSM number	Type of Reconstruction	Incision length (cm)	Tumor size (cm)	Breast tissue weight (g)	Therapeutic NSM	Prophylactic NSM	Bilateral NSM	Follow up (m)
[29]	Italy	Single-center, prospective	73	42 (mean/ median)	20.5 (mean/ median)	94	Implant (89.4%) Expander (10.6%)	N/A	1.7 (mean)	N/A	63.8%	36.2%	N/A	20 (mean)
[37]	Taiwan	Single-center, retrospective	51	48 (mean)	N/A	54	Implant	4 (mean)	3.4	293 (mean)	100%	0%	5.9%	14.6 (mean)
[30]	Italy	Phase III, open- label, single- center RCT	40	44.5 (median)	N/A	51	Implant (88.2%) Expander (11.8%)	N/A	N/A	N/A	N/A	N/A	27.5%	28.6 (median)
[20]	USA	Single-center, prospective	20	40 (median)	24.4 (median)	40	Expander (Prepectoral)	4 (median)	N/A	318 (median)	22.5%	77.5%	100%	Up to 36
[15]	South Korea	Single-center, retrospective	42	43.2 (mean)	23 (mean)	46	Implant	5	N/A	346.4 (mean)	89.1%	10.9%	11.9%	9.4 (mean)
[21]	South Korea	Single-center, retrospective	39	46.63 (median)	N/A	46	Implant (Prepectoral)	N/A	N/A	N/A	N/A	N/A	17.9%	N/A
[13]	Taiwan	Prospective, multi-center, non-randomized trial	66	48.2 (mean)	N/A	76	Implant (Subpectoral)	4.1 (mean)	2.5 (mean)	282.1 (mean)	100%	0%	15.2%	28.4 (mean)
[42]	Taiwan	Single-center, retrospective	233	49.2 (mean)	21.5 (mean)	266	Implant (94.4%) Expander (5.6%)	4	N/A	N/A	89.5%	10.5%	14.2%	37.2 (mean)

Overview of the characteristics of prosthesis-based reconstruction studies.

**Table 2:** The table summarises key features of each study reporting single-incision RNSM with mixed reconstruction cohorts. BMI: body mass index; NSM: nipple-sparing mastectomy; DIEP: deep inferior epigastric perforator; PAP: profunda artery perforator; LD: latissimus dorsi; TRAM: transverse rectus abdominis myocutaneous; N/A: not available; cm: centimeter; g: grams

Study	Location	Study design	Patients’ number	Age in years	BMI	NSM number	Type of Reconstruction	Incision length (cm)	Tumor size (cm)	Breast tissue weight (g)	Therapeutic NSM	Prophylactic NSM	Bilateral NSM	Follow up (months)
[18]	Taiwan	Single-center retrospective	22	44.1 (mean)	24.1 (mean)	22	DIEP flap (84.4%) PAP flap (15.6%)	≤5 in most cases	N/A	350.4 (mean)	100%	0%	0%	12.4 (mean)
[32]	South Korea	Multi-center retrospective	73	45.4 (median)	21.4 (median)	82	Implant (59.8%) Expander (37.8%) DIEP flap (1.2%) LD flap (1.2%)	5 (median)	1.1 (median)	306 (median)	91.5%	8.5%	N/A	N/A
[19]	Taiwan	Single-center prospective	43	47.8 (mean)	22.8 (mean)	54	Implant (96.2%) TRAM flap (1.9%) LD flap (1.9%)	N/A	2.5 (mean)	279 (mean)	88.9%	11.1%	25.6%	49.3 (mean)
[33]	South Korea	single-center retrospective	118	45.9 (mean)	26.3 (mean)	118	Implant (58.5%) Flap (41.5%)	4 (mean)	1.5 (mean)	N/A	100%	0%	0%	Up to 1
[49]	South Korea	single-center retrospective	162	44.8 (mean)	22.2 (mean)	186	Implant (83.9%) DIEP flap (14.2%) LD flap (1.9%)	N/A	N/A	N/A	N/A	N/A	14.8%	24.5 (median)

Overview of the characteristics of mixed cohort studies.

**Table 3:** The table summarises the peri-operative variables of each study. NSM: nipple-sparing mastectomy; mL: millilitre; LOS: length of stay; N/A not available

Study	Unilateral NSM time (minutes)	Blood loss (mL)	LOS (days)	Conversion to open	Reoperation	Positive margins
[29]	N/A	N/A	2	0	4	0
[37]	224	35	7	0	0	1
[18]	216	N/A	13.3	0	2	1
[30]	N/A	202	2.3	0	1	N/A
[32]	354.2	N/A	12	0	2	2
[19]	N/A	41	N/A	N/A	N/A	N/A
[20]	202.4	N/A	N/A	0	3	0
[33]	N/A	N/A	N/A	0	3	7
[15]	N/A	103.7	N/A	N/A	2	N/A
[21]	N/A	N/A	N/A	N/A	4	N/A
[13]	N/A	33.8	5.2	0	N/A	1
[42]	N/A	N/A	3.9	0	7	0
[49]	270.6	N/A	N/A	N/A	N/A	N/A

### Quality assessment

Assessment of methodological quality of the included studies was performed using the Joanna Briggs Institute (JBI) Checklist for Prevalence Studies [23]. This quality assessment method was used since it is more suitable for studies reporting proportional outcomes and prevalence data. The JBI checklist evaluates nine domains, and each study was rated independently across these domains. Judgments were made by two independent reviewers based on information provided in the original publications (Tables S3 and S4). Overall, the quality assessment of the included studies was considered acceptable for this analysis.

### Statistical analysis

The primary outcome was the pooled proportion of the binary outcomes (prevalence). Event rates were transformed using the Freeman–Tukey double-arcsine method, which stabilizes variances [24]. Pooled estimates were calculated under a random-effects model (REML) due to anticipated heterogeneity [24]. Subgroup analyses or meta-regression were not performed because the number of studies reporting each outcome was small, which could result in unreliable or spurious findings [25]. Continuous outcomes (e.g., operative time and blood loss) were calculated using inverse-variance weighting.

Forest plots were generated to visually display individual study proportions and pooled estimates. Study-level 95% confidence intervals, with statistical significance defined as *p* < 0.05, were calculated using the Wilson score, a method that provides accurate coverage and balanced interval width compared to other methods, especially for small samples or extreme proportions [26]. Heterogeneity was quantified via the I^2^ statistic (low: 25%, moderate: 50%, and high: 75%).

Publication bias was visually demonstrated as funnel plots; however, the small number of included studies limits the ability to exclude it [27]. Formal statistical tests for funnel plot asymmetry (e.g., Egger’s test) were not performed because the pooled outcomes were reported from a small number of studies, which may yield unreliable results [28]. Statistical analyses were performed using Stata/BE 19.0 software.

## Results

### Study characteristics

Thirteen studies published between 2019 and 2025 were included, comprising prospective (n = 5) and retrospective (n = 8) designs and presenting 982 patients who underwent 1135 single-incision RNSM procedures across four countries (Italy, Taiwan, South Korea, and the USA). The single-incision RNSM was performed either for therapeutic (87%) or prophylactic (13%) purposes. Approximately 15% of cases were bilateral NSMs. Average patient age ranged from 40 to 49 years, and body mass index (BMI) ranged from 20.5 to 26.3 kg/m^2^. Following the NSM procedure, the majority of cases (92%) underwent prosthesis-based reconstruction (including tissue expander or direct-to-implant), while the remaining smaller proportion involved autologous tissue (flap) reconstruction. The reported single incision was 4–5 cm in length, while the average breast tissue weight ranged between 279 and 350 g. Follow-up durations varied significantly, ranging from 9 to 49 months. Detailed per-study characteristics are summarized in Table 1 and Table 2.

### Peri-operative parameters

**Fig. 2:**
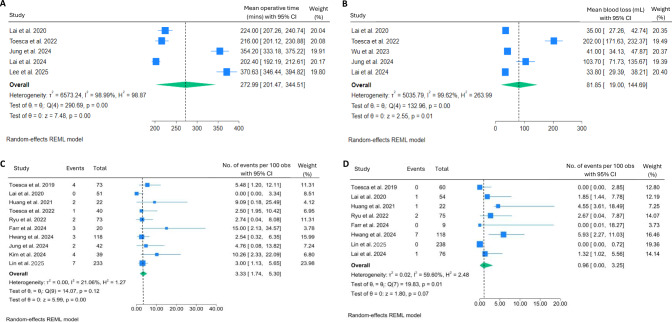
Forest plots showing the pooled results of perioperative parameters: operative time in minutes (**A**), blood loss in mL (**B**), reoperation rate (**C**), and positive margins rate (**D**)

Operative time was reported by most of the included studies. Pooled analysis revealed a mean operative time of 273 min (95% CI: 201.5–344.5, I^2^ = 98.99%, *p* < 0.001). Substantial variation existed among the studies, as the statistical analysis revealed significant heterogeneity (Fig. 2A). This implies that the significant disparities in operative time could be attributed to the diversity in the techniques during surgery or the post-mastectomy reconstruction procedure. Intraoperative blood loss was inconsistently reported, with only five studies quantitatively reporting it. Generally, the estimated blood loss was low, with a pooled result of 82 mL (95% CI: 19.0–144.7, I^2^ = 99.62%, *p* < 0.001) (Fig. 2B).

No conversions to open surgery and no perioperative mortality were reported in the included studies. Reoperation rates were generally low, 3.3% (95% CI: 1.74–5.30, I^2^ = 21.06%, *p* = 0.12) (Fig. 2C), and were mainly performed due to immediate post-operative complications including haematoma, infection, or skin necrosis. Pooled result of positive surgical margins was < 1% (95% CI: 0.00–3.25, I^2^ = 59.60%, *p* = 0.01) (Fig. 2D).

Length of hospital stay (LOS) was reported in 6 out of 13 studies and ranged from 2 to 13.3 days. Shorter LOS was reported in cohorts of patients who underwent post-mastectomy implant/expander placement [13, 29–31], while longer LOS was observed in cohorts of patients who, at least part of them, underwent flap reconstruction [18, 32]. A summary of the perioperative parameters for each study is presented in Table 3.

### Complication profile

**Fig. 3:**
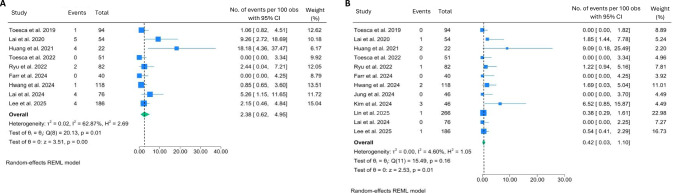
Forest plots showing the pooled results of post-operative complication rates: skin flap necrosis (**A**) and NAC necrosis (**B**)

The overall complication rates were found to be generally low. Skin flap necrosis was observed in 2.4% of the cases (95% CI: 0.62–4.95) with moderate heterogeneity across the studies (I^2^ = 62.87%, *p* = 0.01). The highest skin flap necrosis rate (18.2%) was recorded in the only study that reported a cohort of patients who exclusively underwent post-mastectomy flap reconstruction [18], which appears to be the main contributor to heterogeneity (Fig. 3). On the other hand, necrosis of the nipple-areolar complex (NAC) was observed in a much lower rate, 0.4% of the cases (95% CI: 0.03–1.10; I^2^ = 4.6%, *p* = 0.16) (Fig. 3B).

**Fig. 4:**
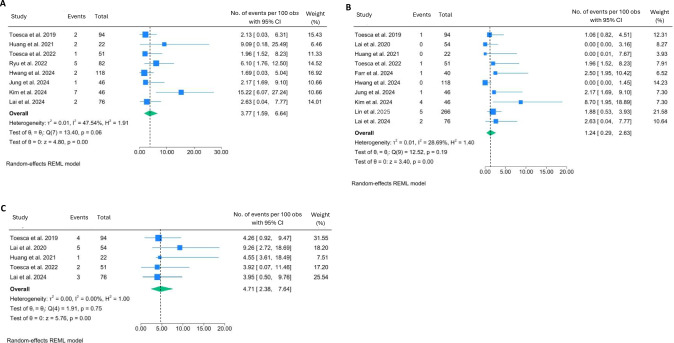
Forest plots showing the pooled results of post-operative complication rates: SSI (**A**), deep infection (**B**), and delayed wound healing (**C**)

The SSI occurred among 3.8% of the patients (95% CI: 1.59–6.64; I^2^ = 47.54%, *p* = 0.06) (Fig. 4A), while deep infection rate, including cases of implant-related infection, was relatively lower at 1.2% (95% CI: 0.29–2.63, I^2^ = 28.7%, *p* = 0.19) (Fig. 4B). Delayed wound healing showed a pooled rate at 4.7% (95% CI: 2.38–7.64; I^2^ = 0.00%, *p* = 0.75) (Fig. 4C).

**Fig. 5:**
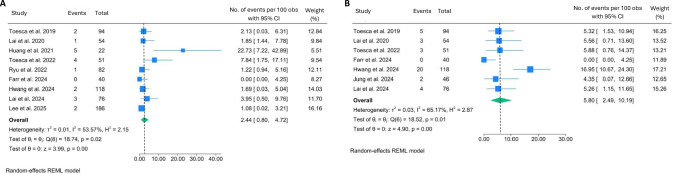
Forest plots showing the pooled results of post-operative complication rates: hematoma (**A**) and seroma (**B**)

Hematoma was recorded in 2.4% (95% CI: 0.80–4.72; I^2^ = 53.57%, *p* = 0.02). The highest hematoma occurrence rate (22.7%) was recorded in the only cohort of patients who exclusively underwent post-mastectomy flap reconstruction [18], which significantly contributed to heterogeneity (Fig. 5A). On the other hand, the occurrence of seroma was relatively higher, with a rate of 5.8% (95% CI: 2.49–10.19; I^2^ = 65.17%, *p* = 0.01). The moderate heterogeneity can be explained, at least in part, by the outlier rate (17%) reported in a cohort of patients in which 41.5% underwent flap reconstruction (Fig. 5B) [33].

**Fig. 6:**
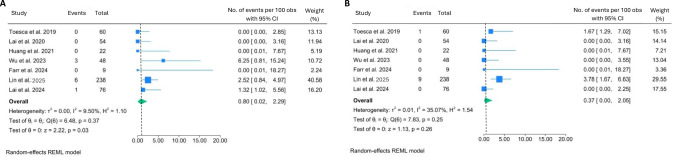
Forest plots showing the pooled results of cancer recurrence rates: LRR (**A**) and distant recurrence (**B**)

Locoregional recurrence (LRR) of cancer occurred in 0.8% (95% CI: 0.02–2.29; I^2^ = 9.5%, *p* = 0.37) (Fig. 6 A), whereas distant recurrence of cancer occurred at a relatively lower rate, 0.4% (95% CI: 0.00–2.05; I^2^ = 35.07%, *p* = 0.25) (Fig. 6B). No study reported procedure-related mortality or unexpected oncologic compromise during follow-up. Funnel plots showed no significant asymmetry for most endpoints, indicating small risks of bias (Figure S1).

## Discussion

This systematic review and single-arm meta-analysis study synthesizes an up-to-date quantitative evidence of single-incision RNSM feasibility and outcomes. This study demonstrates acceptable perioperative parameters, low postoperative complication rates, and favorable oncologic outcomes. The findings in this study demonstrate that single-incision RNSM is a technically feasible and safe procedure. The reported low complication rates of this approach were comparable to or superior to the reported outcomes of the conventional or other minimally invasive NSM approaches.[35, 36]. In addition, the single-incision approach offers cosmetic advantages by restricting the number of incisions to a single incision, particularly when placed laterally. This may enhance patient satisfaction and postoperative outcomes [37].

Ouyang et al. (2025) reported a meta-analysis of outcomes from three studies exclusively utilizing the da Vinci SP system, representing true single-port RNSM [34]. However, the present study is more comprehensive, incorporating all available studies reporting single-incision RNSM performed using various da Vinci platforms (Si, Xi, X, and SP). All procedures were performed through a single axillary incision with a mean length of 4–5 cm, comparable to the true single-port RNSM incision length [34]. In addition, this study distinctively investigates oncologic safety outcomes, including clear surgical margins and cancer recurrence rates.

The pooled total operative time in the single-incision NSM was longer than conventional NSM but consistent with early experiences in robotic-assisted breast surgery [38, 39]. High heterogeneity in the reported operative duration reflects variations in surgeon experience, different operative/robotic settings, case complexity, and reconstructive approach. In one study, operation time gradually decreased as the experience improved from the first to the final cases [39]. With growing proficiency and optimized port access, subsequent studies have reported shortening of the robotic console and docking times as surgical experience accumulates, suggesting a progressive learning curve [40].

Intra-operative blood loss, reoperation rate, and LOS in this study were small, which are comparable to the current evidence derived from large cohorts of conventional and robotic NSM patients [41]. Conversion to open surgery was distinctly not reported in any of the included studies, suggesting an improved learning compared to earlier experiences of RNSM, where conversion to open surgery was marginally above zero [38, 39]. The positive surgical margins rate was less than 1%, a remarkably lower rate compared to recent evidence from robotic (4%) and conventional (7%) NSM [42].

The single-incision RNSM has reported a low overall rate of post-operative complications. Skin flap and NAC necrosis rates were significantly lower compared to conventional and other minimally invasive NSM approaches (e.g., endoscopic and multi-port robotic approaches) [35, 36]. Likewise, infection rates, including superficial and deep SSI, and delayed wound healing, were relatively lower [35, 36]. Hematoma and seroma both occurred in single-incision NSM at rates comparable to those recorded in conventional and other robotic NSM [43]. The LRR and distant recurrence of cancer rates are substantially lower than those observed with conventional procedures [44, 45]; however, the heterogeneity in follow-up durations across the included studies warrants cautious interpretation of these findings.

Several limitations associated with this analysis must be acknowledged. This is a single-arm meta-analysis which limits definitive conclusions regarding the relative effectiveness compared to other approaches. Most of the included studies had retrospective designs with small sample sizes [15, 18, 21], with heterogeneity in reporting peri-operative and post-operative outcomes. Long-term oncologic/recurrence outcomes remain underreported in approximately half of the included studies. In addition, most of the included studies reported mixed cohorts without separate complication profile data for different patient populations, such as the indication for surgery (prophylactic versus therapeutic mastectomy), precluding reliable subgroup analysis. Future prospective studies should therefore consider reporting indication- and approach-specific outcomes to enable meaningful comparative assessments. Another limitation is that most of the pooled outcomes in this analysis were derived from less than 10 studies, limiting the reliability of formal statistical testing for publication bias. Consequently, publication bias was assessed through visual inspection of funnel plots, and the results should be interpreted with appropriate caution. The use of an English-language filter may also introduce selection bias. These limitations introduce potential biases related to reporting and selection [46]. Although the individual studies have small sample sizes that limit generalisability, this meta-analysis of proportions is well-suited for synthesizing evidence across these studies, as it stabilizes variance and enables a more accurate estimation of prevalence [47].

The high heterogeneity observed for some outcomes likely reflects different surgeons’ experience, operative and robotic settings, and post-mastectomy immediate reconstruction procedures across the included studies [48]. Given the limited number of studies contributing to each outcome, formal subgroup analyses or meta-regression were not undertaken, and the pooled prevalence estimates should therefore be interpreted with caution [25]. Assessment of publication bias was limited to visual inspection of funnel plots. Quantitative tests, such as Egger’s regression, were not applied due to the small number of studies per outcome and substantial heterogeneity, which may compromise the validity of such tests [28].

Nonetheless, this study offers important insights. The consistency of findings across diverse geographical and temporal settings suggests that single-incision RNSM provides a reproducible and generally acceptable treatment approach for breast cancer treatment or prophylactic intervention. Future research should focus on prospective, comparative, and cost-effectiveness trials comparing single-incision RNSM with multi-port robotic, endoscopic, and conventional (open) NSM approaches. Standardized reporting of cosmetic, functional, and oncologic endpoints is essential to validate the reproducibility and scalability of this innovative method.

## Conclusion

Within the limitations of this study, the single-incision approach in RNSM represents a safe and feasible minimally invasive surgical approach with low morbidity and promising reconstructive outcomes. Although current evidence is encouraging, further high-quality studies are required to reliably investigate the subgroup comparison, long-term oncologic safety, economic value, and learning curve dynamics.

## Supplementary Information

Below is the link to the electronic supplementary material.

Supplementary file1 (PDF 436 KB)

Supplementary file2 (DOCX 19 KB)

Supplementary file3 (DOCX 20 KB)

Supplementary file4 (DOCX 25 KB)

Supplementary file5 (DOCX 23 KB)

## Data Availability

This study used data derived from published literature. No new primary data were generated.

## References

[1] Rusby JE, Smith BL, Gui GPH (2010) Nipple-sparing mastectomy. Br J Surg 97:305–316. 10.1002/bjs.6970 20101646 10.1002/bjs.6970

[2] Ashikari AY, Kelemen PR, Tastan B, Salzberg CA, Ashikari RH (2018) Nipple sparing mastectomy techniques: a literature review and an inframammary technique. Gland Surg 7:273–287. 10.21037/gs.2017.09.02 29998077 10.21037/gs.2017.09.02 PMC6006015

[3] Wei CH, Scott AM, Price AN, Miller HC, Klassen AF, Jhanwar SM et al (2016) Psychosocial and sexual well-being following nipple-sparing mastectomy and reconstruction. Breast J 22:10–17. 10.1111/tbj.12542 26782950 10.1111/tbj.12542 PMC4843778

[4] Romanoff A, Zabor EC, Stempel M, Sacchini V, Pusic A, Morrow M (2018) A comparison of patient-reported outcomes after nipple-sparing mastectomy and conventional mastectomy with reconstruction. Ann Surg Oncol 25:2909–2916. 10.1245/s10434-018-6585-4 29968023 10.1245/s10434-018-6585-4 PMC6205203

[5] Hallbeck MS, Law KE, Lowndes BR, Linden AR, Morrow M, Blocker RC et al (2020) Workload differentiates breast surgical procedures: NSM associated with higher workload demand than SSM. Ann Surg Oncol 27:1318–1326. 10.1245/s10434-019-08159-0 31916090 10.1245/s10434-019-08159-0 PMC7138769

[6] Parks L (2021) Nipple-sparing mastectomy in breast cancer: impact on surgical resection, oncologic safety, and psychological well-being. J Adv Pract Oncol 12:499–506. 10.6004/jadpro.2021.12.5.5 34430060 10.6004/jadpro.2021.12.5.5 PMC8299789

[7] Toesca A, Peradze N, Galimberti V, Manconi A, Intra M, Gentilini O et al (2017) Robotic nipple-sparing mastectomy and immediate breast reconstruction with implant: first report of surgical technique. Ann Surg 266:e28-30. 10.1097/SLA.0000000000001397 28692558 10.1097/SLA.0000000000001397

[8] Branco T, Rodrigues A, Martins AR (2025) Robotics in breast surgery: current advantages, disadvantages, and applications. Cureus 17:e87972. 10.7759/cureus.87972 40821160 10.7759/cureus.87972 PMC12354885

[9] la De Cruz-Ku G, Chambergo-Michilot D, Perez A, Valcarcel B, Pamen L, Linshaw D et al (2023) Outcomes of robotic nipple-sparing mastectomy versus conventional nipple-sparing mastectomy in women with breast cancer: a systematic review and meta-analysis. J Robot Surg 17:1493–1509. 10.1007/s11701-023-01547-5 36808041 10.1007/s11701-023-01547-5

[10] Nessa A, Shaikh S, Fuller M, Masannat YA, Kastora SL (2024) Postoperative complications and surgical outcomes of robotic versus conventional nipple-sparing mastectomy in breast cancer: meta-analysis. Br J Surg. 10.1093/bjs/znad336 37890072 10.1093/bjs/znad336 PMC10769157

[11] Maes-Carballo M, Gómez-Fandiño Y, Braña PMS, Martínez-Martínez C, Alberca-Remigio C, Cámara-Martínez C et al (2025) Robotic nipple-sparing mastectomy: a comparative analysis with conventional and endoscopic techniques through a systematic review. J Robot Surg 19:220. 10.1007/s11701-025-02388-0 40377824 10.1007/s11701-025-02388-0

[12] Doll A, Kopkash K, Baker J (2024) Emerging role of robotic surgery in the breast. Clin Breast Cancer 24:286–291. 10.1016/j.clbc.2023.12.009 38220537 10.1016/j.clbc.2023.12.009

[13] Lai H-W, Chen D-R, Liu L-C, Chen S-T, Kuo Y-L, Lin S-L et al (2024) Robotic Versus Conventional or Endoscopic-assisted Nipple-sparing Mastectomy and Immediate Prosthesis Breast Reconstruction in the Management of Breast Cancer: A Prospectively Designed Multicenter Trial Comparing Clinical Outcomes, Medical Cost, and Patient-reported Outcomes (RCENSM-P). Ann Surg 279:138–146. 10.1097/SLA.0000000000005924 37226826 10.1097/SLA.0000000000005924 PMC10727200

[14] Sarfati B, Honart J-F, Leymarie N, Rimareix F, Al Khashnam H, Kolb F (2018) Robotic da Vinci Xi-assisted nipple-sparing mastectomy: first clinical report. Breast J 24:373–376. 10.1111/tbj.12937 29251382 10.1111/tbj.12937

[15] Jung SM, Kim YJ, Lee K-T, Jeon B-J, Mun G-H, Pyon J-K et al (2024) Learning curve for robot-assisted nipple-sparing mastectomy: a single institution experience. Eur J Surg Oncol 50:108602. 10.1016/j.ejso.2024.108602 39167863 10.1016/j.ejso.2024.108602

[16] Park HS, Kim JH, Lee DW, Song SY, Park S, Kim SI et al (2018) Gasless robot-assisted nipple-sparing mastectomy: a case report. J Breast Cancer 21:334–338. 10.4048/jbc.2018.21.e45 30275863 10.4048/jbc.2018.21.e45 PMC6158155

[17] Go J, Ahn JH, Park JM, Choi SB, Lee J, Kim JY et al (2022) Analysis of robot-assisted nipple-sparing mastectomy using the da Vinci SP system. J Surg Oncol 126:417–424. 10.1002/jso.26915 35622078 10.1002/jso.26915

[18] Huang J-J, Chuang EY-H, Cheong DC-F, Kim B-S, Chang FC-S, Kuo W-L (2021) Robotic-assisted nipple-sparing mastectomy followed by immediate microsurgical free flap reconstruction: feasibility and aesthetic results – case series. Int J Surg 95:106143. 10.1016/j.ijsu.2021.106143 34666195 10.1016/j.ijsu.2021.106143

[19] Wu W-P, Lai H-W, Liao C-Y, Lin J, Huang H-I, Chen S-T et al (2023) Use of magnetic resonance imaging for evaluating residual breast tissue after robotic-assisted nipple-sparing mastectomy in women with early breast cancer. Korean J Radiol 24:640–646. 10.3348/kjr.2022.0708 37404106 10.3348/kjr.2022.0708 PMC10323414

[20] Farr DE, Haddock NT, Tellez J, Radi I, Alterio R, Sayers B et al (2024) Safety and feasibility of single-port robotic-assisted nipple-sparing mastectomy. JAMA Surg 159:269–276. 10.1001/jamasurg.2023.6999 38231502 10.1001/jamasurg.2023.6999 PMC10794977

[21] Kim SH, Park S, Lee DW, Park HS, Lew DH, Song SY (2024) Early experience of direct-to-implant breast reconstruction using acellular dermal matrix after robot-assisted nipple-sparing mastectomy. Plast Reconstr Surg 154:512–520. 10.1097/PRS.0000000000011105 37797243 10.1097/PRS.0000000000011105

[22] Page MJ, Moher D, Bossuyt PM, Boutron I, Hoffmann TC, Mulrow CD et al (2021) PRISMA 2020 explanation and elaboration: updated guidance and exemplars for reporting systematic reviews. BMJ 372:n160. 10.1136/bmj.n160 33781993 10.1136/bmj.n160 PMC8005925

[23] Munn Z, Moola S, Lisy K, Riitano D, Tufanaru C (2015) Methodological guidance for systematic reviews of observational epidemiological studies reporting prevalence and cumulative incidence data. Int J Evid Based Healthc 13:147–153. 10.1097/XEB.0000000000000054 26317388 10.1097/XEB.0000000000000054

[24] Nyaga VN, Arbyn M, Aerts M (2014) Metaprop: a Stata command to perform meta-analysis of binomial data. Arch Public Health 72:39. 10.1186/2049-3258-72-39 25810908 10.1186/2049-3258-72-39 PMC4373114

[25] Spineli LM, Pandis N (2020) Problems and pitfalls in subgroup analysis and meta-regression. Am J Orthod Dentofacial Orthop 158:901–904. 10.1016/j.ajodo.2020.09.001 33250104 10.1016/j.ajodo.2020.09.001

[26] Liu X, Liu F, Liu W, Huang Y, Li Z, Wu Q et al (2025) Sample size planning for proportions based on Wilson score confidence intervals with precision and assurance. Commun Stat Theory Methods 0:1–15. 10.1080/03610926.2025.2560629

[27] Afonso J, Ramirez-Campillo R, Clemente FM, Büttner FC, Andrade R (2024) The perils of misinterpreting and misusing “publication bias” in meta-analyses: an education review on funnel plot-based methods. Sports Med 54:257–269. 10.1007/s40279-023-01927-9 37684502 10.1007/s40279-023-01927-9 PMC10933152

[28] Sterne JAC, Sutton AJ, Ioannidis JPA, Terrin N, Jones DR, Lau J et al (2011) Recommendations for examining and interpreting funnel plot asymmetry in meta-analyses of randomised controlled trials. BMJ 343:d4002. 10.1136/bmj.d4002 21784880 10.1136/bmj.d4002

[29] Toesca A, Invento A, Massari G, Girardi A, Peradze N, Lissidini G et al (2019) Update on the feasibility and progress on robotic breast surgery. Ann Surg Oncol 26:3046–3051. 10.1245/s10434-019-07590-7 31342391 10.1245/s10434-019-07590-7 PMC7493284

[30] Toesca A, Sangalli C, Maisonneuve P, Massari G, Girardi A, Baker JL et al (2022) A randomized trial of robotic mastectomy versus open surgery in women with breast cancer or BrCA mutation. Ann Surg 276:11–19. 10.1097/SLA.0000000000004969 34597010 10.1097/SLA.0000000000004969

[31] Lin Y-MC, Lui SA, Chen M-Y, Chou Y-Y, Cheng FT-F (2025) Safety and feasibility of robotic nipple-sparing mastectomy with immediate direct-to-implant reconstruction – insights from the one of the largest centers in Asia. Clin Breast Cancer 25:277–282. 10.1016/j.clbc.2024.12.013 39824711 10.1016/j.clbc.2024.12.013

[32] Ryu JM, Kim JY, Choi HJ, Ko B, Kim J, Cho J et al (2022) Robot-assisted nipple-sparing mastectomy with immediate breast reconstruction: an initial experience of the Korea Robot-endoscopy Minimal Access Breast Surgery Study Group (KoREa-BSG). Ann Surg 275:985–991. 10.1097/SLA.0000000000004492 32941285 10.1097/SLA.0000000000004492

[33] Hwang Y-H, Han HH, Eom JS, Yoo T-KR, Kim J, Chung IY et al (2024) Evaluation of safety and operative time in tumescent-free robotic nipple-sparing mastectomy: a retrospective single-center cohort study. Ann Surg Treat Res 107:8–15. 10.4174/astr.2024.107.1.8 38978689 10.4174/astr.2024.107.1.8 PMC11227914

[34] Ouyang R, Jia X, Liang Y, Li B, Qing M, Hu J (2025) Single-port robotic nipple-sparing mastectomy: a systematic review and single-arm meta-analysis of safety and process outcomes. J Robot Surg 20:106. 10.1007/s11701-025-03074-x 41428248 10.1007/s11701-025-03074-x

[35] Kim JH, Ryu JM, Bae SJ, Ko BS, Choi JE, Kim KS et al (2024) Minimal access vs conventional nipple-sparing mastectomy. JAMA Surg 159:1177–1186. 10.1001/jamasurg.2024.2977 39141399 10.1001/jamasurg.2024.2977 PMC11325243

[36] Elghazaly S, Fakeh S, Elbarbary S, Mahmoud K, Hilali AE, Gamal P et al (2025) Robot-assisted versus open surgery in early-stage breast cancer: a systematic review and meta-analysis. Clin Breast Cancer. 10.1016/j.clbc.2025.08.019 40962684 10.1016/j.clbc.2025.08.019

[37] Lai H-W, Chen S-T, Mok CW, Chang Y-T, Lin S-L, Lin Y-J et al (2021) Single-port three-dimensional (3D) videoscope-assisted endoscopic nipple-sparing mastectomy in the management of breast cancer: technique, clinical outcomes, medical cost, learning curve, and patient-reported aesthetic results from 80 preliminary procedures. Ann Surg Oncol 28:7331–7344. 10.1245/s10434-021-09964-2 33934239 10.1245/s10434-021-09964-2

[38] Toesca A, Peradze N, Manconi A, Galimberti V, Intra M, Colleoni M et al (2017) Robotic nipple-sparing mastectomy for the treatment of breast cancer: feasibility and safety study. Breast 31:51–56. 10.1016/j.breast.2016.10.009 27810700 10.1016/j.breast.2016.10.009 PMC5278881

[39] Houvenaeghel G, Bannier M, Rua S, Barrou J, Heinemann M, Van Troy A et al (2019) Breast cancer robotic nipple sparing mastectomy: evaluation of several surgical procedures and learning curve. World J Surg Oncol 17:27. 10.1186/s12957-019-1567-y 30728011 10.1186/s12957-019-1567-y PMC6366058

[40] Rademacher N, Curwick LA, Parker CC (2025) A brief history and the current state of robotic mastectomy: a review. Curr Breast Cancer Rep 17:31. 10.1007/s12609-025-00587-0 40655393 10.1007/s12609-025-00587-0 PMC12254071

[41] Kim CW, Yoo T-K, Kim J, Chung I-Y, Ko BS, Kim HJ et al (2025) Postoperative outcomes of single-port robot-assisted versus conventional nipple-sparing mastectomy with immediate reconstruction. Ann Surg Oncol. 10.1245/s10434-025-18733-4 41233708 10.1245/s10434-025-18733-4

[42] Kuo W-L, Huang J-J, Chu C-H, Chang S-C, Lin Y-J, Chuang Y-H et al (2025) Comparative analysis of oncological and surgical outcomes of robotic versus conventional mastectomy for breast cancer. Eur J Surg Oncol. 10.1016/j.ejso.2025.109622 39884089 10.1016/j.ejso.2025.109622

[43] Nessa A, Shaikh S, Fuller M, Masannat YA, Kastora SL (2023) Postoperative complications and surgical outcomes of robotic versus conventional nipple-sparing mastectomy in breast cancer: meta-analysis. Br J Surg. 10.1093/bjs/znad336 10.1093/bjs/znad336 PMC10769157 37890072

[44] De La Cruz L, Moody AM, Tappy EE, Blankenship SA, Hecht EM (2015) Overall survival, disease-free survival, local recurrence, and nipple-areolar recurrence in the setting of nipple-sparing mastectomy: a meta-analysis and systematic review. Ann Surg Oncol 22:3241–3249. 10.1245/s10434-015-4739-1 26242363 10.1245/s10434-015-4739-1

[45] Poruk KE, Ying J, Chidester JR, Olson JR, Matsen CB, Neumayer L et al (2015) Breast cancer recurrence after nipple-sparing mastectomy: one institution’s experience. Am J Surg 209:212–217. 10.1016/j.amjsurg.2014.04.001 24946727 10.1016/j.amjsurg.2014.04.001

[46] Althubaiti A (2016) Information bias in health research: definition, pitfalls, and adjustment methods. J Multidiscip Healthc 9:211–217. 10.2147/JMDH.S104807 27217764 10.2147/JMDH.S104807 PMC4862344

[47] Barendregt JJ, Doi SA, Lee YY, Norman RE, Vos T (2013) Meta-analysis of prevalence. J Epidemiol Community Health 67:974–978. 10.1136/jech-2013-203104 23963506 10.1136/jech-2013-203104

[48] Pluta P, Rathat G, Blay L, Gentilini OD, Huber DE, Daniel M et al (2025) Minimal access nipple-sparing mastectomy – the current European landscape. Prz Menopauzalny 24:66–71. 10.5114/pm.2025.150082 40718010 10.5114/pm.2025.150082 PMC12288482

[49] Lee J, Go J, Lee SJ, Kwon Y, Kim NH, Kim JY et al (2025) Comparative study of mastectomy using conventional techniques, multiport and single-port robotic surgical systems. Cancer Res Treat. 10.4143/crt.2025.115 40340261 10.4143/crt.2025.115 PMC13093034

