# Supplementary material for: Feasibility and outcomes of single-incision robotic nipple-sparing mastectomy: a systematic review and meta-analysis
Source: J Robot Surg. 2026 Mar 11;20(1):341. doi: 10.1007/s11701-026-03297-6 (PMC12975829; doi:10.1007/s11701-026-03297-6)
Supplement: Supplementary file 1 — Supplementary file1 (PDF 436 KB) [file 11701_2026_3297_MOESM1_ESM.pdf]

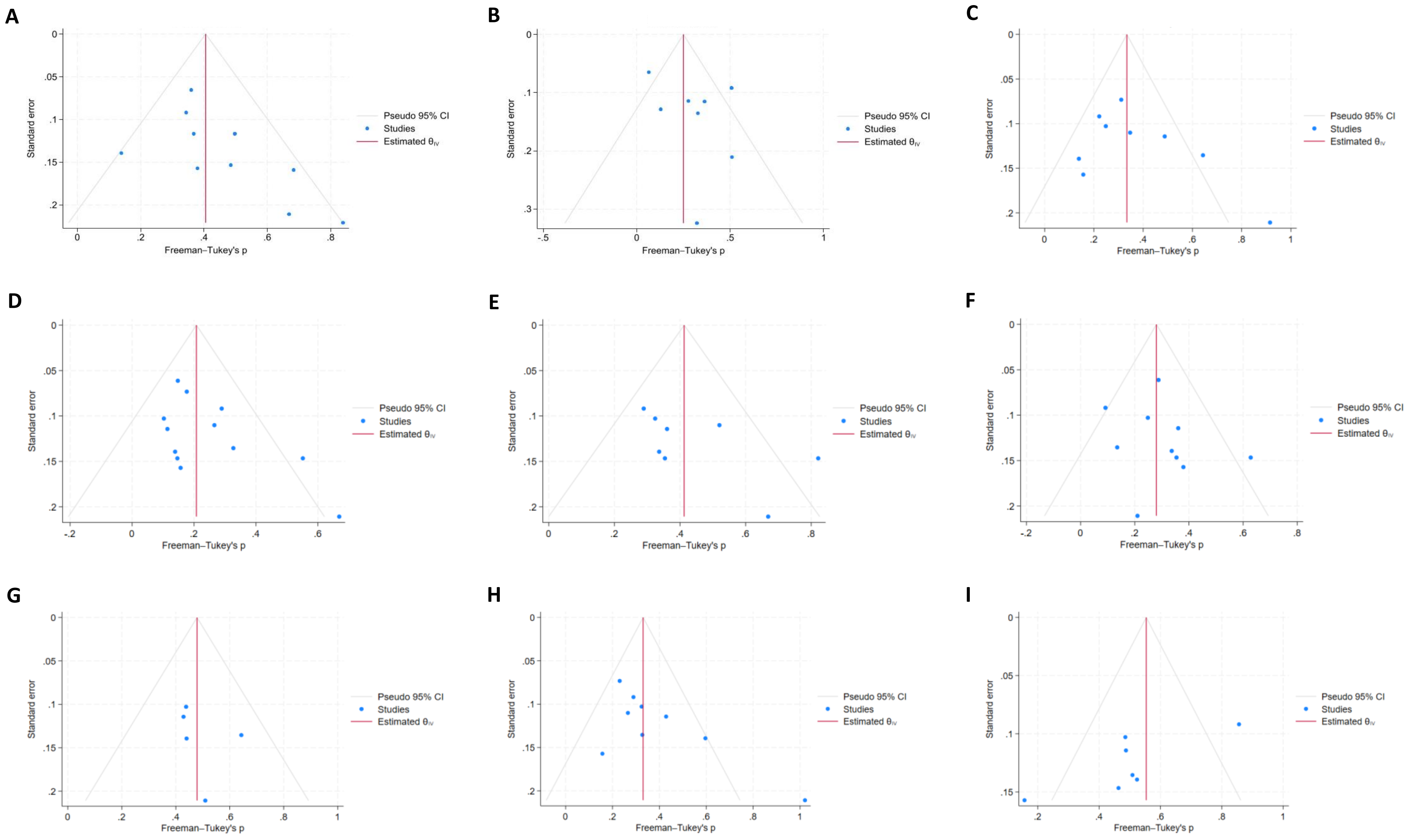
A
B
C
D
E
F
G
H
I
Standard error
Freeman–Tukey's p
Pseudo 95% CI
Studies
Estimated θ_{IV}

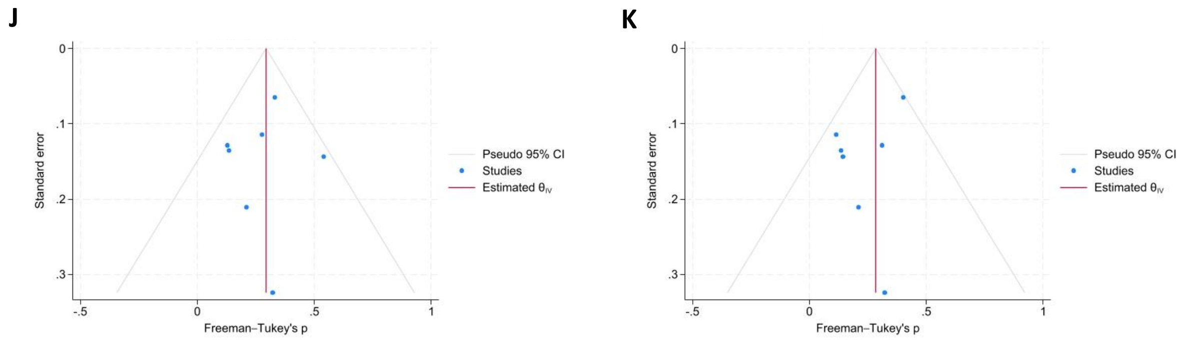

Figure 6. Funnel plots assessing publication bias for reoperation (A), positive margins (B), skin flap necrosis (C), NAC necrosis (D), SSI (E), deep infection (F), delayed healing (G), hematoma (H), seroma (I), LRR (J), and distant cancer recurrence (K).
