# Supplementary material for: Feasibility and outcomes of single-incision robotic nipple-sparing mastectomy: a systematic review and meta-analysis
Source: J Robot Surg. 2026 Mar 11;20(1):341. doi: 10.1007/s11701-026-03297-6 (PMC12975829; doi:10.1007/s11701-026-03297-6)
Supplement: Supplementary file 2 — Supplementary file2 (DOCX 19 KB) [file 11701_2026_3297_MOESM2_ESM.docx]

Search strategy employed across databases

| **Database** | **Search strategy** |
| --- | --- |
| PubMed | ((single?port OR "single incision" OR robotic OR "robot?assisted" OR endoscopic) AND ("nipple?sparing mastectomy" OR NSM))[tiab] |
| Medline (Ovid) | ((single?port or "single incision" or robotic or "robot?assisted" or endoscopic) and ("nipple?sparing mastectomy" or NSM)).ti,ab. |
| Scopus | (TITLE-ABS((single?port or "single incision" or robotic or "robot?assisted" or endoscopic) and ("nipple?sparing mastectomy" or NSM)) |
| Web of science | (single?port or "single incision" or robotic or "robot?assisted" or endoscopic) (Title) and ("nipple?sparing mastectomy" or NSM) (Title) OR (single?port or "single incision" or robotic or "robot?assisted" or endoscopic) (Abstract) and ("nipple?sparing mastectomy" or NSM) (Abstract) |

**Table S1:** Search strategy employed across four databases (PubMed, Medline (Ovid), Scopus, Web of Science). Search strategies included relevant keywords and Boolean operators to capture relevant studies.
