# Supplementary material for: Feasibility and outcomes of single-incision robotic nipple-sparing mastectomy: a systematic review and meta-analysis
Source: J Robot Surg. 2026 Mar 11;20(1):341. doi: 10.1007/s11701-026-03297-6 (PMC12975829; doi:10.1007/s11701-026-03297-6)
Supplement: Supplementary file 3 — Supplementary file3 (DOCX 20 KB) [file 11701_2026_3297_MOESM3_ESM.docx]

**The PICO framework for study design, eligibility criteria, and outcomes**

| **Component** | **Description** |
| --- | --- |
| **Population (P)** | Adult female patients undergoing nipple-sparing mastectomy for therapeutic or prophylactic indications. |
| **Intervention (I)** | Single-incision or single-port robotic nipple-sparing mastectomy. |
| **Comparator (C)** | None (single-arm meta-analysis). |
| **Outcomes (O)** | Operative time, blood loss, reoperation, margin clearance, length of stay, individual postoperative complications, and cancer recurrence. |

**Table S2:** A table summarizing the study design, eligibility criteria of the included records, and measured outcomes according to the PICO framework (Population, Intervention, Comparator, and Outcomes).
