# Supplementary material for: Feasibility and outcomes of single-incision robotic nipple-sparing mastectomy: a systematic review and meta-analysis
Source: J Robot Surg. 2026 Mar 11;20(1):341. doi: 10.1007/s11701-026-03297-6 (PMC12975829; doi:10.1007/s11701-026-03297-6)
Supplement: Supplementary file 5 — Supplementary file5 (DOCX 23 KB) [file 11701_2026_3297_MOESM5_ESM.docx]

Quality assessment of the included studies by the second reviewer.

| JBI Item | Toesca et al. 2019 | Lai et al. 2020 | Huang et al. 2021 | Toesca et al. 2022 | Ryu et al. 2022 | Wu et al. 2023 | Farr et al. 2024 | Hwang et al. 2024 | Jung et al. 2024 | Kim et al. 2024 | Lin et al.2025 | Lai et al. 2024 | Lee et al. 2025 |
| --- | --- | --- | --- | --- | --- | --- | --- | --- | --- | --- | --- | --- | --- |
| 1. Was the sample frame appropriate to address the target population? | Yes | Yes | Yes | Yes | Yes | Yes | Yes | Yes | Yes | Yes | Yes | Yes | Yes |
| 2. Were study participants recruited in an appropriate way? | Yes | Yes | Yes | Yes | Yes | Yes | Yes | Yes | Yes | Yes | Yes | Yes | Yes |
| 3. Was the sample size adequate? | Yes | Yes | Unclear | Yes | Yes | Yes | Unclear | Yes | Yes | Unclear | Yes | Yes | Yes |
| 4. Were the study subjects and setting described in detail? | Yes | Yes | Yes | Yes | Yes | Unclear | Yes | Unclear | Unclear | Unclear | Yes | Yes | Unclear |
| 5. Was the data analysis conducted with sufficient coverage of the identified sample? | Yes | Yes | Yes | Yes | Yes | Unclear | Yes | Yes | Yes | Yes | Yes | Yes | Yes |
| 6. Were valid methods used for the identification of the condition? | Yes | Yes | Yes | Yes | Yes | Unclear | Yes | Yes | Yes | Yes | Yes | Yes | Yes |
| 7. Was the condition measured in a standard, reliable way for all participants? | Yes | Yes | Yes | Yes | Yes | Unclear | Yes | Yes | Yes | Unclear | Unclear | Yes | Unclear |
| 8. Was there appropriate statistical analysis? | Yes | Yes | Yes | Yes | Yes | Yes | Yes | Yes | Yes | Yes | Yes | Yes | Yes |
| 9. Was the response rate adequate, and if not, was the low response rate managed appropriately? | Unclear | N/A | N/A | Unclear | N/A | Unclear | Unclear | N/A | N/A | N/A | N/A | Unclear | N/A |

**Table S4:** Quality assessment of included studies using the Joanna Briggs Institute (JBI) Checklist for Prevalence Studies, as conducted by the second reviewer. Each item of the checklist was evaluated across all included studies, with responses recorded as “Yes,” “Unclear,” “N/A” (not applicable), or “No.”
